# On the Treatment of External Hemorrhoids by Potassa Fusa

**Published:** 1850-03

**Authors:** 


*On the Treatment of External Hemorrhoids by Potassa Fusa.* By Dr. F. C. Jones, Surgeon to the South London Dispensary.

Dr. Jones states that within twelve months he has treated between sixty and seventy cases of external piles with undeviating success, by the means which he here describes. He says:

Observing that in all cases of spontoneous cure of external hemorrhoids, they primarily slough, I was led to think that if nature obliterates the vessels by sloughing, it is feasible that artificial means might induce the same remedial action.

In turning over in my mind the action of various remedies likely to produce this condition, it struck me forcibly that potassa fusa was precisely the agent required. I forthwith determined to apply it to the first case that came under my care, and the results were, that upon the first application the patient complained of a burning sensation, which passed off in the course of half an hour; at the end of four days the parts were considerably diminished in size, and by brushing a piece of lint quickly over the part I removed the superficial slough, and again applied the potassa fusa; the same treatment was followed at the interval of four days, at the end of which time they had entirely disappeared, leaving only a slight sore, which healed within a week.—*Lancet, Sept.* 8, 1849.

